# Partial cellular reprogramming: A deep dive into an emerging rejuvenation technology

**DOI:** 10.1111/acel.14039

**Published:** 2023-12-01

**Authors:** Patrick T. Paine, Ada Nguyen, Alejandro Ocampo

**Affiliations:** ^1^ Department of Biomedical Sciences, Faculty of Biology and Medicine University of Lausanne Lausanne Vaud Switzerland; ^2^ Center for Virology and Vaccine Research Harvard Medical School Boston Massachusetts USA; ^3^ Zygo Bio San Francisco California USA; ^4^ EPITERNA SA Epalinges Switzerland; ^5^ Present address: McGovern Institute for Brain Research at MIT, Massachusetts Institute of Technology Cambridge Massachusetts USA

**Keywords:** aging, aging hallmarks, lifespan, partial cellular reprogramming, rejuvenation

## Abstract

Aging and age‐associated disease are a major medical and societal burden in need of effective treatments. Cellular reprogramming is a biological process capable of modulating cell fate and cellular age. Harnessing the rejuvenating benefits without altering cell identity via partial cellular reprogramming has emerged as a novel translational strategy with therapeutic potential and strong commercial interests. Here, we explore the aging‐related benefits of partial cellular reprogramming while examining limitations and future directions for the field.

## Graphical abstract

Partial cellular reprogramming is able to improve or restore multiple age‐related phenotypes including lifespan, healthspan, epigenetic age, aging hallmarks, and tissue regeneration.

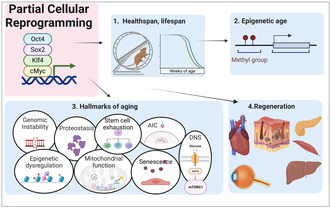

**Abbreviations**
AAD: age‐associated diseases
HGPS: hutchinson gilford progeria syndrome
IVPR: in vivo partial reprogramming
OSKM: Oct4, Sox2, Klf4, cMyc

## 1 AGE‐RELATED DISEASES

Significant increases in lifespan worldwide have shifted demographics dramatically over the last 100 years to the point; there is now an estimated 125 million people 80 years and older. Elderly individuals over 65 years currently outnumber those under 5 years old, demonstrating a “graying” of the human population (Partridge et al., 2020). The unintended consequence of this increase in lifespan has been the exponential growth of age‐associated disease that accompany aging (Garmany et al., 2021). Although more people can now expect to live longer, their healthspan, also known as healthy life expectancy, shows little or no improvement based on morbidity and disability (Crimmins, 2015). The top four age‐associated diseases (AADs) including cardiovascular diseases, cancer, respiratory diseases, and diabetes now account for more than 2/3 of all mortality annually worldwide with related health care costs in the trillions of dollars (Beard et al., 2016; Nugent, 2019). On top of this, in 2015, the World Health Organization announced the rise of AADs as a global epidemic (Kassebaum et al., 2017). It is now clear that significant improvement to healthspan and treatment of aging and AADs represents an important medical and societal goal but success requires a clear understanding of the aging processes involved.

## 2 AGING PHENOMENA

Ordinarily, molecular and cellular aging experienced by living organisms is counteracted through evolutionarily derived processes including for example the DNA damage repair response or autophagy (Ferrucci et al., 2010; Tian et al., 2017). The process of aging results in a progressive failure in these critical repair and homeostatic mechanisms subsequently inducing a decline in physiological function, increased susceptibility to disease, and eventual mortality (Rando & Chang, 2012). In order to describe and quantify this process, several categories of aging drivers, the so‐called hallmarks of aging, have been identified including epigenetic alterations, genomic instability, loss of proteostasis, telomere attrition, deregulated nutrient sensing, mitochondrial dysfunction, cellular senescence, stem cell exhaustion, and altered intercellular communication (López‐Otín et al., 2013). Recently, additional hallmarks have been proposed including cell size alterations, stochastic non‐enzymatic modification of long lived molecules, and dysbiosis (Davies et al., 2022; Fedintsev & Moskalev, 2020; López‐Otín et al., 2023). Inclusion as a hallmark of aging requires that the phenotype manifests during aging, and if manipulated can accelerate aging or slow down its progress. Although these age sensitive phenotypes are important readouts to enable identification of novel interventions, the underlying processes, mechanisms, and potential interrelationships to which they contribute are still under investigation (Keshavarz et al., 2022).

August Weismann first noted over a hundred years ago the immortal germline exists in a privileged state, uncoupled from aging, in order to transmit hereditary information across generations while somatic cells lack this selective pressure and are therefore finite (Larocca et al., 2021; West et al., 2019). In this line, the somatic restriction theory of aging proposes that aging in multicellular animals is a result of the confinement of pluripotent traits, like unlimited self‐renewal, to the germ line (West et al., 2019). It is not until these cells exit the pluripotent state and differentiate into somatic cells that aging ensues, resulting in replicative senescence (Hayflick & Moorhead, 1961). In multicellular organisms, the global aging process is currently proposed to be a multi‐causal and interrelated phenomenon^9^. Indeed, the majority of programmed and damage‐related aging theories are not mutually exclusive and are often proposed to overlap by modern theorists (Bjorksten, 1968; Gladyshev, 2016; Harman, 1992; Jin, 2010; Kane & Sinclair, 2019; Kirkwood, 2005; Szilard, 1959; Weismann, 1893; West et al., 2019; Williams, 2001).

## 3 CELLULAR REPROGRAMMING

In the mid‐20th century, the epigenetic landscape model was first proposed to describe the unidirectional process of development following oocyte fertilization (Waddington, 1957). John Gurdon overturned this concept when he first demonstrated nuclear reprogramming. Specifically, he showed a cell fate could be reversed into a viable zygote via the transfer of an adult intestinal epithelial cell nucleus into an oocyte using somatic cell nuclear transfer (SCNT; Gurdon, 1962). This process of converting a somatic cell into a dedifferentiated toti‐ or pluripotent cell is called cellular reprogramming (Saitou et al., 2012). During development, cellular reprogramming occurs following fertilization and is driven by epigenetic remodeling, reorganization of the chromatin state, extinguishment of the somatic identity, and finally expression of the pluripotent gene regulatory network (Koche et al., 2011; Morgan et al., 2005; Polo et al., 2012). Importantly, as cell identity is reset, all age accrued defects are restored and a minimal biological age, referred to as ground zero, is reached during early embryogenesis (Kerepesi et al., 2021; Seisenberger et al., 2013). Confirmation of these profound observations are demonstrated in cloning experiments. Specifically, clones produced with adult somatic cells via SCNT are born young, with restored telomere length, live a full lifespan, and can be serially cloned further, suggesting complete rejuvenation has occurred (Lanza et al., 2000; Sinclair et al., 2016; Wakayama et al., 2013; Wilmut et al., 1997). Still, SCNT is technically challenging and reliant on endogenous maternal factors and chromatin modifying enzymes within the egg to replace the somatic proteins and machinery to induce reprogramming (Jullien et al., 2011). Importantly, this process was streamlined in 2006 when Shinya Yamanaka's laboratory screened 24 genes associated with embryonic stem cell identity and discovered four transcription factors; Oct4, Sox2, Klf4, and cMyc (OSKM) sufficient to induce pluripotency in mouse fibroblasts (Takahashi & Yamanaka, 2006). Together, Gurdon and Yamanaka were awarded the 2012 Nobel Prize for the discovery of cellular reprogramming and thus significantly advanced research into the field of regenerative medicine.

Initial investigations into the age‐related effects of cellular reprogramming using pluripotent transcription factors in vitro demonstrated a restoration of telomere size, gene expression profile, oxidative stress, and mitochondrial metabolism in both human centenarian fibroblasts and senescent cells (Lapasset et al., 2011; Lee et al., 2020; Yagi et al., 2012). Still, important questions remained, namely, can the restoration of cellular age be separated from the loss of cell fate. A solution to this problem, first proposed by Singh and Zacouto (2010), presented the concepts of partial reprogramming and partial cloning in which reprogramming would be induced only until the cells had undergone signs of rejuvenation. These ideas were later confirmed by Manukyan and Singh (2014) when they first observed epigenetic rejuvenation in senescent human fibroblasts following 9 days of reprogramming without loss of cell identity. Specifically, 5 factor expression (Oct 4, Sox 2, Klf4, cMyc, and Lin28) via a piggybac transposase vector restored the mobility of heterochromatin protein 1 (HP1) to young levels without progression through a dedifferentiated state (Manukyan & Singh, 2014). Follow‐up work has since demonstrated that the dose of reprogramming is linked to the amount of age rejuvenation. Notably, when aged human cells are reprogrammed for up to 20 days, the epi‐age based on the DNA methylation clock is completely reverted to zero suggesting a dose dependent effect (Olova et al., 2019). By demonstrating that the loss of cell identity due to cellular reprogramming could be separated from the impacts on aging, the next stage was set for translational investigations in vivo.

## 4 IN VIVO PARTIAL REPROGRAMMING

**FIGURE 1:**
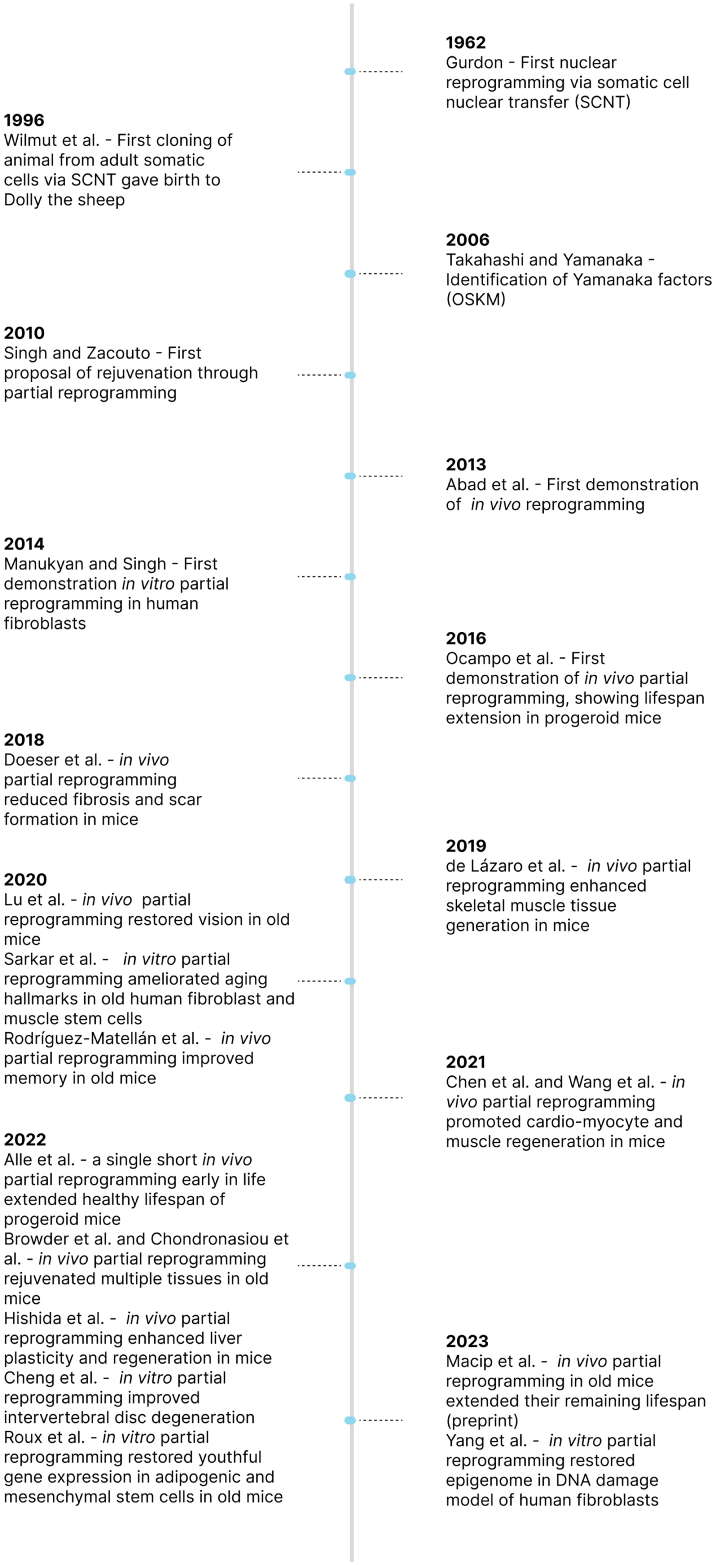
Timeline of key partial reprogramming studies. Key historical milestones in the development of partial reprogramming for rejuvenation beginning with John Gurdon first demonstrating nuclear reprogramming is possible using the somatic cell nuclear transfer technique.

**FIGURE 2:**
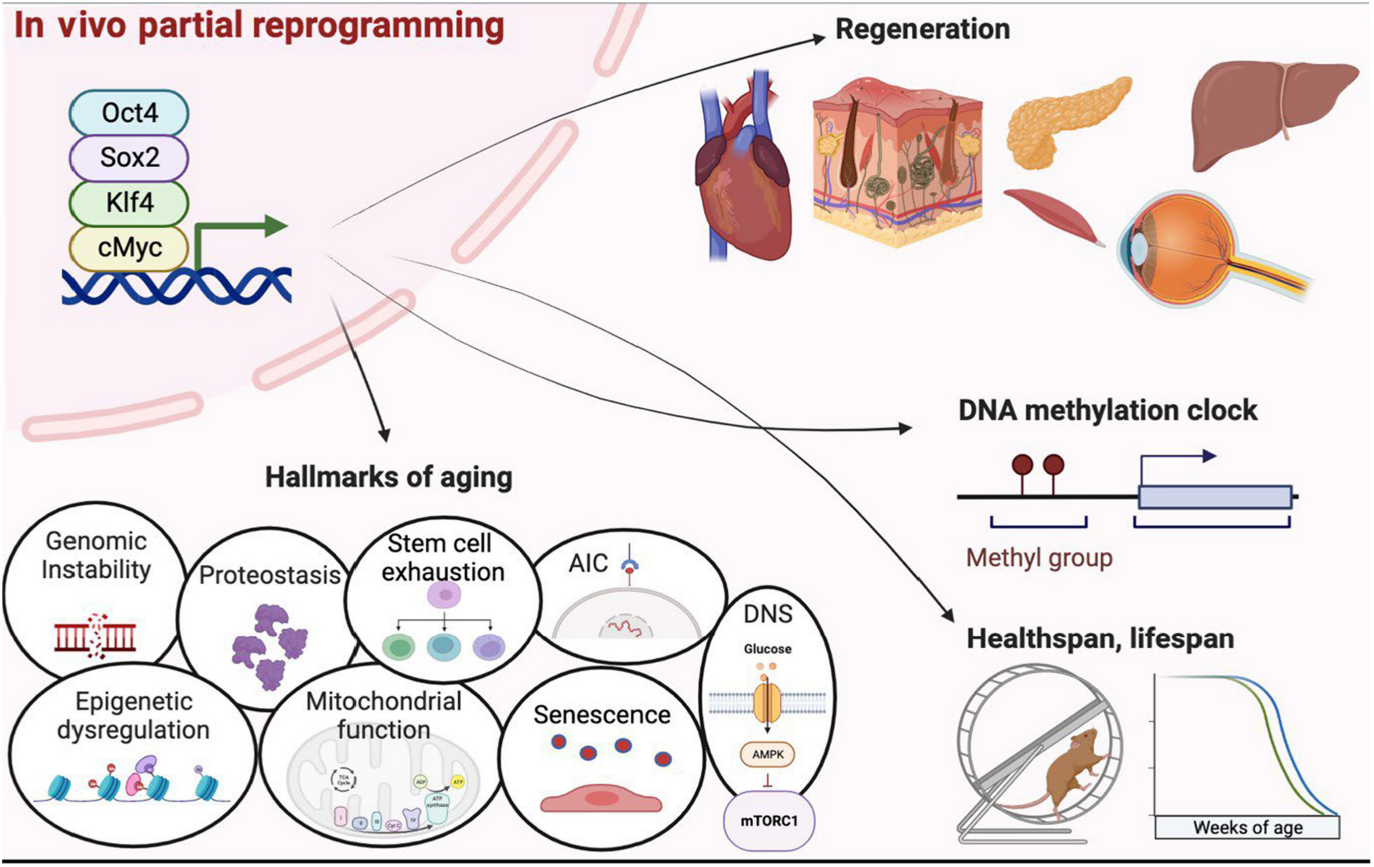
Multiparameter rejuvenation following in vivo partial reprogramming (IVPR). In vivo partial reprogramming drives rejuvenated phenotypes in tissue repair and function, hallmarks of aging, DNAm clock, and extends healthspan and lifespan. (created with biorender.com).

In vivo cellular reprogramming via OSKM expression provides an important strategy to investigate cellular reprogramming based phenomena, including regeneration and aging, within a biologically relevant multicellular organism. Initial attempts at in vivo reprogramming using a mouse model with a doxycycline inducible OSKM transgene cassette generated pluripotent cells capable of forming the three germ layers and mouse chimeras with germline contribution. At the same time, full pluripotency induction in vivo proved problematic due to dysplastic cell proliferation and teratoma formation across multiple organs (Abad et al., 2013; Ohnishi et al., 2014). To avoid any deleterious oncogenic changes associated with acquisition of pluripotency, a novel treatment strategy was developed using a short term and cyclic induction protocol called in vivo partial reprogramming (IVPR). IVPR in mice successfully prevented loss of cell identity while still extending lifespan and ameliorating multiple hallmarks of aging (Ocampo, Reddy, Martinez‐Redondo, et al., 2016). Since this important proof of principle, multiple laboratories have further demonstrated that IVPR can increase lifespan and restore tissue dysfunction across multiple tissues and organs including the dentate gyrus, optic nerve, liver, skeletal muscle, skin, intervertebral disc, and heart, although with some limitations and hurdles to yet be overcome (Figure 1; Alle et al., 2022; Chen et al., 2021; Cheng et al., 2022; de Lázaro et al., 2019; Hishida et al., 2022; Lu et al., 2020; Rodríguez‐Matellán et al., 2020). In order to validate future clinical development, here we review the key partial reprogramming studies, both in vivo and in vitro, in detail as related to lifespan, epigenetic age, hallmarks of aging, and regenerative effects (Figure 2).

## 5 LIFESPAN EXTENSION

Lifespan extension is one of the key readouts to demonstrate the effects of anti‐aging interventions based on chronological time. Several groups have tested the benefits of IVPR on lifespan with varying results (Table 1). At this point, two groups have observed an increase in median and maximum lifespan following IVPR in a short‐lived LAKI progeria model of premature aging. The LAKI mouse is an accelerated model of aging with a truncated lamin A protein, called progerin, which is responsible for Hutchinson Gilford progeria syndrome in humans (HGPS; Osorio et al., 2011).

**TABLE 1:** Lifespan.

Model	Partial reprogramming protocol	Lifespan increase	Ref
LAKI (homozygous) 4F mice	2 days on, 5 days off, starting at 2 months old until death	33% median 18% maximum	Ocampo, Reddy, Martinez‐Redondo, et al. (2016)
LAKI (heterozygous) 4F mice	2 days on, 5 days off, starting at 2 months old until death	26% median 16% maximum	Alle et al. (2022)
LAKI (heterozygous) 4F mice	Single short induction, starting at 2 months old for 2.5 weeks	No change median 18% maximum	Alle et al. (2022)
Wildtype 4F mice	Single short induction, starting at 2 months old for 2.5 weeks	No change median No change maximum 13% increase third quartile	Alle et al. (2022)
Wildtype mice	1 week on, 1 week off, starting at 124 weeks old until death	109% increase in remaining median lifespan	Macip et al. (2023)

Abbreviation: 4F, 4 factors.

In 2016, seminal work from Ocampo, Reddy, Martinez‐Redondo, et al. (2016) first demonstrated that IVPR, via a cyclic induction of OSKM (2 days on and 5 days off), could extend median and maximum lifespan in a homozygous LAKI mouse model by 33% and 18%, respectively. Alternatively, the Lemaitre group tested the effects of IVPR in both wildtype mice and heterozygous LAKI mice with a less severe progeroid phenotype as an intermediary model (Alle et al., 2022). First, they explored dosage effects in the LAKI mouse using either a high dose of doxycycline cyclically or a low dose continuously beginning at 2 months of age. Both approaches resulted in a similar increase in median and maximal lifespan. At the same time, they documented that a single short burst of OSKM induction with an intermediate dose of doxycycline in 8‐week‐old LAKI mice significantly increased the third quartile lifespan by almost 10 weeks and maximal lifespan by 11 weeks. Potentially, this single burst of IVPR was effective due to a deeper alteration of the epigenome that can occur after 2.5 weeks of reprogramming (Alle et al., 2022; Gill et al., 2022). In line with this observation, Macip et al. (2023) observed a significant effect of partial reprogramming on late stage median lifespan when applied to aged wild type mice. Using an adeno‐associated virus 9 (AAV9) vector to systemically deliver OSK to 124‐week‐old mice, the group showed that IVPR could extend the remaining median lifespan of treated mice by 109% (Macip et al., 2023).

These improvements to the lifespan of homozygotic and heterozygotic LAKI mice along with wild type mice following IVPR are important demonstrations of reprogramming induced rejuvenation. Notably, the benefits are most robust in the most severe premature aging model perhaps due to a rescue of the complex pathology specific to the homozygous mutant (Ocampo, Reddy, & Belmonte, 2016). A key limitation to the current research using transgenic mouse models is the variability in the expression of the reprogramming factors, and therefore the total amount of reprogramming, among different cells, tissue types, and organs (Alle et al., 2021; Chondronasiou et al., 2022; Parras et al., 2022). In this line, it has been demonstrated that different cell types reprogram at different rates and follow specific reprogramming trajectories (Hussein et al., 2014; Nefzger et al., 2017; Tonge et al., 2014; Zunder et al., 2015). For this reason, whole‐body OSKM induction is confounded by excess reprogramming and loss of function in some cells while under reprogramming without rejuvenation in others. This could impact lifespan studies in several ways, for example, a loss of digestive function and therefore an unintended caloric restriction effect could overstate reprogramming effects. In fact, improved whole‐body reprogramming models are an ongoing pursuit and at the same time have shifted emphasis into tissue‐specific models of reprogramming, targeted reprogramming, or enhanced reprogramming methods (Chen et al., 2021; de Lázaro et al., 2019; Hishida et al., 2022; Lu et al., 2020; Parras et al., 2022; Roux et al., 2022; Wang et al., 2021).

## 6 EPIGENETIC AGE

The ability to determine epigenetic age based on gain or loss of methylation at specific CpG loci is a recent important discovery for accurately measuring biological age in cells and tissues (Hannum et al., 2013; Horvath, 2013). In vitro experiments demonstrated the epigenetic age of fully reprogrammed aged cells can be completely restored to zero (Horvath, 2013; Olova et al., 2019). In addition, several groups (Table 2) have now shown an improvement to epigenetic age following partial reprogramming in vivo and in vitro using mouse and human cells (Browder et al., 2022; Chondronasiou et al., 2022; Gill et al., 2022; Lu et al., 2020; Sarkar et al., 2020; Yang et al., 2023). Recently, the Belmonte group observed that in 15‐month‐old wildtype mice given 7 months of IVPR, 2 days on and 5 days off, the epigenetic clock was improved in the kidney and skin but not the liver, lung, muscle, or spleen (Browder et al., 2022). Simultaneously, they observed no improvement to the epigenetic clock in 25‐month‐old wildtype mice following IVPR for 1 month within those tissues. In line with this, Chondronasiou et al. (2022) observed rejuvenation of differentially methylated promoters and enhancers in wildtype 55‐week‐old mice pancreas, liver, and the spleen, after only 1 week of IVPR. Lu et al. (2020) performed an intra‐ocular injection with an AAV vector containing 3 factors; Oct 4, Sox 2, and Klf4 (OSK) and observed a decrease in DNA methylation age based on ribosomal DNA in retinal ganglion cells from 12‐month‐old wildtype mice after 4 weeks of continuous reprogramming. Using a unique double strand break accelerated aging mouse model, Yang et al. (2023) also showed this 3 factor AAV vector reversed epigenetic age in fibroblasts up to 57% based on four different epigenetic clocks. The Sebastiano group pioneered a novel IVPR technique using modified RNA (modRNA) of 6 pluripotency factors including Oct 4, Sox 2, cMyc, Klf 4, Lin 28, and Nanog. Transient reprogramming with this 6F modRNA cocktail for 4 days in vitro restored epigenetic and transcriptomic age in old human fibroblasts and endothelial cells in vitro (Sarkar et al., 2020). Gill et al. (2022) observed aged human fibroblasts transduced with a 4F cassette underwent a morphological shift associated with a mesenchymal to epithelial transition and restored epigenetic age during reprogramming. Specifically, they observed 13 days of reprogramming reduced median DNA methylation clock age by approximately 30 years and was superior to 10, 15, or 17 days of reprogramming (Gill et al., 2022).

**TABLE 2:** Epigenetic age.

Model	Partial reprogramming protocol	Clock type	Epigenetic age	Ref
Human Fb (60–90 yo)	modRNA delivery of OSKMLN, in vitro for 4 days	Horvath pan‐tissue clock. Skin and blood clock	−1.84 years, −1.07 years	Sarkar et al. (2020)
Human EC (50–65 yo)	mRNA delivery of OSKMLN, in vitro for 4 days	Horvath pan‐tissue clock. Skin and blood clock	−4.94 years, −1.62 years	Sarkar et al. (2020)
RGC 12‐month‐old WT mice	intra‐orbital injection of AAV OSK, in vivo for 4 weeks	Ribosomal DNA methylation clock	Significant reduction	Lu et al. (2020)
MEFs DNA damage model	In vitro AAV OSK, induced for 10 days	4 clocks: Thompson, Petkovich, Meer, and Stubbs	Up to 57% decrease	Yang et al. (2023)
Human Fb (38–53 yo)	lentiviral delivery of OSKM, in vitro for 13 days	Horvath pan‐tissue clock, Skin and blood clock	−30 years	Gill et al. (2022)
Mouse WT 4F 12‐month‐old, 15‐month‐old	In vivo OSKM, 2 days on 5 days off, for 10 months	Lifespan Uber Correlation clock	Significant reduction in skin and kidney no changes in liver, lung, muscle, spleen	Browder et al. (2022)
Mouse WT 4F 25‐month‐old	In vivo OSKM, 2 days on 5 days off, for 1 months	Lifespan Uber Correlation clock	No change	Browder et al. (2022)
Mouse WT 4F 55‐week‐old	In vivo low dose OSKM, 1 week burst	No clock. Methylation profile	Restored in pancreas, liver, and spleen	Chondronasiou et al. (2022)

Abbreviations: AAV, adeno‐associated virus; C, chondrocyte; EC, endothelial cell; Fb, fibroblast; LV, lentivirus; NPC, nucleus pulposus cells; OSKMLN, Oct4, Sox2, Klf4, cMyc, Lin28, Nanog; RGC, retinal ganglion cell; SC, satellite cells.

In summary, multiple laboratories have shown reprogramming can induce reversal of epigenetic aging in both mice and human cells, in vivo and in vitro, and across multiple cell types although with some caveats. Browder et al. (2022) for example saw improvement in several tissues, but not all, following IVPR in wildtype mice even though expression of the 4Fs was similar. Browder also did not see reduction to the epigenetic clock in aged mice after 1 month of IVPR (Browder et al., 2022). At the same time, in vitro data demonstrated improvements to the epigenetic age with partial reprogramming but individual results varied and in several specific instances no restoration was observed (Sarkar et al., 2020). Possible reasons for these discrepancies could be due to the low efficiency of reprogramming, irreversibly aged phenotypes in specific tissues, variance in rates of epigenetic aging, or inherent differences in reprogramming potential due to cell type of origin and environmental cues (Browder et al., 2022; Gill et al., 2022; Nefzger et al., 2017). Notably, the results produced by an epigenetic clock analysis are strongly dependent on the tissue type and the clock selected, suggesting the alterations to cellular identity during reprogramming could be confounding at times (Liu et al., 2020).

## 7 AGING HALLMARKS

Currently, partial reprogramming has been demonstrated to effectively ameliorate 8 of 9 hallmarks of aging, although specific results vary depending on the assay, protocol, and model used (Table 3). Interestingly, some hallmarks of aging in different models are improved following a short treatment of 2 or 4 days while others require a more substantial reprogramming duration. As a key driver of aging, it is notable that DNA damage shows improvement following short‐term reprogramming in fibroblasts of wildtype and progeria mouse models along with nucleus pulposus cells from naturally aged mice (Cheng et al., 2022; Chondronasiou et al., 2022; Ocampo, Reddy, Martinez‐Redondo, et al., 2016). Epigenetic dysregulation is a critical driver of aging according to the Information Loss Theory of aging (Kane & Sinclair, 2019). Cellular reprogramming has been shown to improve a variety of epigenetic markers including the DNA methylome, H3K9me3, H4K20me3, H3k36me2, HP1a, and nuclear abnormalities (Browder et al., 2022; Cheng et al., 2022; Chondronasiou et al., 2022; Lu et al., 2020; Manukyan & Singh, 2014; Ocampo, Reddy, Martinez‐Redondo, et al., 2016; Rodríguez‐Matellán et al., 2020; Sarkar et al., 2020; Yang et al., 2023). Improvements to proteostasis after partial reprogramming have been observed based on increased autophagic flux or proteasomal enzyme activity in wildtype mouse and human fibroblasts (Alle et al., 2021; Sarkar et al., 2020). Mitochondrial dysregulation appears restored after partial cellular reprogramming in human and mouse models based on decreased levels of reactive oxygen species, mitochondrial membrane function, and mitochondrial number (Alle et al., 2021; Cheng et al., 2022; Sarkar et al., 2020). Intercellular communication alterations that occur with aging have been ameliorated following reprogramming as demonstrated by decreased inflammaging markers and senescent associated secretory proteins in human chondrocytes and mouse wildtype and progeria models (Alle et al., 2021; Cheng et al., 2022; Chondronasiou et al., 2022; Doeser et al., 2018; Ocampo, Reddy, Martinez‐Redondo, et al., 2016; Sarkar et al., 2020). A decrease in senescence has been demonstrated following short‐term reprogramming based on lower levels of senescence‐associated beta‐Galactosidase (SABG) staining and decreased expression of p16, p21, and p53 in both human and mouse cell types (Alle et al., 2022; Browder et al., 2022; Cheng et al., 2022; Ocampo, Reddy, Martinez‐Redondo, et al., 2016; Sarkar et al., 2020; Wang et al., 2021). Notably, improvement to telomere attrition is a hallmark of aging that remains unimproved with IVPR. This is expected since the increase in telomere length does not appear until late stage reprogramming during conversion to pluripotency (Marión et al., 2017).

**TABLE 3:** Effects of partial reprogramming on aging hallmarks.

Model	Protocol	Cell, tissue	Hallmark of aging ameliorated								Ref
			Ref tissue gen	Epi	Prot	Mito	Nutr	Sen	Stem	Inter	
WT mouse	OSKML, 9 days in vitro	Senescent Fb		Yes							Manukyan and Singh (2014)
4F LAKI Mouse	In vitro, 2+4 day	Fb	Yes	Yes	–	Yes	–	Yes	–	–	Ocampo, Reddy, Martinez‐Redondo, et al. (2016)
6F A Human	In vitro, 4 days	EC, Fb, Ch, SC	Yes	Yes	Yes	Yes	Yes	yes	Yes	Yes	Sarkar et al. (2020)
4F Mouse	In vitro, 2+4 day	NPC, Intervertebral disc	Yes	Yes	–	Yes	Yes	Yes	Yes	–	Cheng et al. (2022)
4F topical Mouse	In vivo, pre‐, post‐injury	Skin	–	–	–	–	–	No	–	–	Doeser et al. (2018)
4F Mouse	In vivo, 4m	Brain	–	Yes	–	–	–	–	–	–	Rodríguez‐Matellán et al. (2020)
3F AAV Mouse	In vivo	RGC, Optic nerve,	–	Yes	–	–	–	–	–	–	Lu et al. (2020)
4F LV Human	In vitro, 10‐13 day	Fb	–	Yes	–	–	–	–	Yes		Roux et al. (2022)
4F Mouse	In vivo, cyclic, 2.5 week	Multi‐tissue	–	Yes	–	Yes	Yes	Yes	Yes	Yes	Browder et al. (2022)
4F Mouse	In vivo, 1 week	Multi‐tissue	–	Yes	–	–	Yes	–	–	Yes	Alle et al. (2022)
4F Mouse	In vivo, 1 week	Liver, spleen, pancreas	No	Yes	–	–	–	No	–	–	Chondronasiou et al. (2022)
3F AAV mouse (ICE)	In vivo, in vitro	Fb, liver, kidney	–	Yes	–	–	–	–	–	–	Yang et al. (2023)

Abbreviations: AAV, adeno‐associated virus; Ch, chondrocyte; EC, endothelial cell; Epi, epigenetic dysregulation; Gen, genomic instability; ICE, inducible changes to the epigenome; Inter, altered intercellular communication. Fb, fibroblast; LV, lentivirus; Mito, mitochondrial dysfunction; NPC, nucleus pulposus cells; Nutri, dysregulated nutrient sensing; Prot, loss of proteostasis; RGC, retinal ganglion cell; SC, satellite cells; Sen, senescence; Stem, stem cell exhaustion.

There are a few exceptions that indicate the amelioration of aging hallmarks via IVPR are context dependent. For example, senescent marker genes were not improved after 1 week of IVPR nor in a skin wound healing model (Chondronasiou et al., 2022; Doeser et al., 2018). In another example, ROS levels were improved in human fibroblasts and chondrocytes but not in endothelial cells (Sarkar et al., 2020). A key technical limitation to some HOA readouts is due to a reprogramming induced decrease in cell and nucleus size (Samavarchi‐Tehrani et al., 2010; van den Hurk et al., 2016). For certain epigenetic changes, this is potentially a confounding factor for observed signal increases based solely on immunostaining and likely requires further validation (Mattout et al., 2011). Generally, these data support a case for IVPR being an effective and reproducible intervention for the majority of key hallmarks of aging with future work needed to address the associated mechanisms that drive the rejuvenated phenotypes.

## 8 REGENERATION AND FUNCTION

Impaired tissue regeneration and fibrosis are well defined aging characteristics with a large clinical burden (Rando & Jones, 2021). In early mammalian development, scarless wound healing occurs until the late gestational fetus and then is lost (Bullard et al., 2003). Interestingly, in some highly regenerative vertebrates including salamanders and fish, wound repair follows a process of dedifferentiation and proliferation mimicking aspects of cellular reprogramming (Jopling et al., 2010; Wang & Simon, 2016). Currently, improvements to proliferation, regeneration, and/or fibrosis following reprogramming have been observed in multiple organs and associated cell types including the skin, muscle, optic nerve, heart, liver, lung, brain, and pancreas (Table 4; Browder et al., 2022; Chen et al., 2021; de Lázaro et al., 2019; Doeser et al., 2018; Guo et al., 2018; Hishida et al., 2022; Lu et al., 2020; Ocampo, Reddy, Martinez‐Redondo, et al., 2016; Sarkar et al., 2020; Seo et al., 2016; Wang et al., 2021).

**TABLE 4:** Effects of partial reprogramming on regeneration.

Tissue	Regeneration	Phenotypes	Function	Model	Protocol	Ref
Muscle	Yes	Increase Pax7 cells	–	4F WT Mouse	Intramuscular injection	Ocampo, Reddy, Martinez‐Redondo, et al. (2016)
Muscle	Yes	Increase proliferation	Increase tetanic force	6F mRNA Ms, Hu	Ex vivo SC	Sarkar et al. (2020)
Muscle	Yes	Increase proliferation, decrease fibrosis	Non‐significant increase to tetanic force	4F plasmid Mouse	In situ	de Lázaro et al. (2019)
Muscle	Yes	Increase Pax7 cells	Grip strength in 2‐4mo	4F myofiber specific Mouse	In vivo, pre‐, post‐ injury	Wang et al. (2021)
Skin	Yes	Increase proliferation, decrease fibrosis	–	4F Mouse	Topical	Doeser et al. (2018)
Skin	Yes	Decrease fibrosis, Increase epidermal proliferation and thickness	–	4F Mouse	In vivo	Browder et al. (2022)
Liver	Yes	Increased proliferation	Increased survival after lethal dose acetaminophen	4F liver specific mouse	In vivo	Hishida et al., 2022
Optic nerve	Yes	Enhanced RGC survival	Improved vision in 11mo after 4wk trx	OSK AAV Mouse	In vivo,	Lu et al. (2020)
Heart	Yes	Increased proliferation, reduced scar	Improved contractile force pre and during injury	4F heart specific Mouse	In vivo, 1 week	Chen et al. (2021)
Lung	Yes	Reduced fibrosis in bleomycin model of pulmonary fibrosis	Improved mechanical function	4F alveolar epithelial cells	Ex vivo, 2 week on, 2 week off	Guo et al. (2018)
Brain	Yes	Increase proliferation, neovascularization	Restored motor function in cerebral ischemia model	4F, mouse	In situ induction	Seo et al. (2016)
Pancreas	Yes	Increase pancreatic islets	Improved glucose tolerance test	4F WT Mouse	In vivo	Ocampo, Reddy, and Belmonte (2016), Ocampo, Reddy, Martinez‐Redondo, et al. (2016)

Abbreviation: RGC, retinal ganglion cell.

As an example, loss of muscle mass, decline in function, and decreased muscle stem cell potency is observed with aging (Blau et al., 2015; Naranjo et al., 2017). Although four groups have demonstrated partial reprogramming can improve muscle regeneration, specific results varied depending on the method of induction and model tested (de Lázaro et al., 2019; Ocampo, Reddy, Martinez‐Redondo, et al., 2016; Sarkar et al., 2020; Wang et al., 2021). For example, Sarkar et al. demonstrated enhanced regeneration and muscle stem cell potency following cardiotoxin injury and transplantation with partially reprogrammed satellite cells from aged humans (60–80 year) or aged mice (20–24 month; Sarkar et al., 2020). At the same time, muscle function based on tetanic force was also restored with this 6 factor modRNA ex vivo method in aged mouse cells (20–24 month; Sarkar et al., 2020). In contrast, muscle regeneration and function was improved in a myofiber specific but not a satellite cell specific transgenic model of 4 factor IVPR in adult wildtype mice (12–15 month; Wang et al., 2021). Previously, it was shown that IVPR with a systemic 4 factor cassette enhanced muscle regeneration and satellite cell proliferation in adult wildtype mice (12 month) after 3 weeks of cyclic induction via intramuscular injection of doxycycline (Ocampo, Reddy, Martinez‐Redondo, et al., 2016). Oppositely, whole‐body cyclic IVPR for 7 months did not significantly improve regeneration in 22 month wild type mice perhaps due to low factor expression (Browder et al., 2022). Interestingly, in situ reprogramming with a 4 factor plasmid vector enhanced regeneration and proliferation with decreased fibrosis following muscle injury to young adult mice (8 week) but cell specific contributions were undetermined (de Lázaro et al., 2019). These results support IVPR as a viable therapeutic route to accelerate muscle repair and function albeit with method and model dependent effects.

Alternatively, skin reprogramming is another example where multiple groups have demonstrated improvements to regeneration and age‐related phenotypes (Alle et al., 2022; Browder et al., 2022; Doeser et al., 2018; Ocampo, Reddy, Martinez‐Redondo, et al., 2016). An Increase in rate of proliferation in skin epidermal cells has been observed following 4F IVPR in both aged wildtype and LAKI mice (Browder et al., 2022; Ocampo, Reddy, Martinez‐Redondo, et al., 2016). Associated increases to epidermal thickness were noted in wild type (female) along with increased dermal thickness in the progeria model (Browder et al., 2022; Ocampo, Reddy, Martinez‐Redondo, et al., 2016). At the same time, improved regeneration capacity by in situ IVPR via local application of doxycycline to an excisional wound model demonstrated decelerated wound closure, reduced fibrosis and scarring, along with a decrease in fibrosis associated TGFb1 expression (Doeser et al., 2018). In wild type aged mice, long‐term IVPR reduced fibrosis and increased epidermal cell proliferation in an excisional wound model, in line with previous results (Browder et al., 2022).

Other individual examples of enhanced regeneration have been noted in several tissues including the liver, optic nerve, heart, and pancreas (Chen et al., 2021; Hishida et al., 2022; Lu et al., 2020; Ocampo, Reddy, Martinez‐Redondo, et al., 2016). In the liver, regenerative capacity and hepatocyte proliferation is enhanced with IVPR following a normally lethal dose of acetaminophen (Hishida et al., 2022). Using an AAV2 vector to deliver OSK directly to retinal ganglion cells (RGCs), Lu et al. (2020) first demonstrated IVPR was able to regenerate the optic nerve and improve RGC survival in a crush injury model. Short‐term cardiomyocyte (CM) specific OSKM expression significantly improved heart regeneration and stimulated CM proliferation following myocardial infarction (Chen et al., 2021). Lastly, in wild‐type aged mice, the size of pancreatic isles and glucose tolerance were increased with IVPR following injury with the beta cell toxin SZT (Ocampo, Reddy, Martinez‐Redondo, et al., 2016). Importantly, improvements to tissue functionality have also been demonstrated in the intervertebral disc, dentate gyrus, kidney, bone, and lung (Alle et al., 2022; Cheng et al., 2022; Rodríguez‐Matellán et al., 2020). In sum, the data show a promising, albeit, context and tissue dependent regenerative effect of partial reprogramming likely confounded at times due to multiple issues including timing, delivery, and dosage along with cell type specific effects of the reprogramming trajectory.

## 9 COMMERCIAL LANDSCAPE

Novel therapeutics able to successfully treat age‐associated chronic disease while restoring the underlying aged phenotype hold immense value for human society. It is estimated that increasing healthspan in the elderly by just 1 year is valued at over 38 trillion dollars (Scott et al., 2021). In the last few years, numerous companies have been established to explore the use of partial reprogramming for the treatment of AADs (Table 5). Among them, Altos Labs stands out with a $3 billion investment from tech investor Yuri Milner and Amazon founder Jeff Bezos, easily making it the largest biotech startup investment in history (Fleming, 2023). Despite the size of investment and number of companies involved, IVPR is still far from being commercially viable. All of the companies listed in Table 5 are currently in preclinical stage, and only Turn Biotechnologies has announced near‐term clinical trials. As the Yamanaka factors are pro‐oncogenic with an associated risk of tumor formation, their therapeutic applications are currently limited and will require improvements to partial reprogramming methods and safety prior to significant expansion of the commercial sector (Klimczak, 2015).

**TABLE 5:** Partial reprogramming in commercial development.

Company	Founded	Funding
Calico	2013	3.5B^a^
Shift Bioscience	2017	<10M
Rejuvenate Bio	2017	17M^a^
Turn Biotechnologies	2018	<20M
Reverse Bioengineering (AgeX subsidiary)	2019	63M^a^
Iduna Therapeutics (Life Biosciences subsidiary)	2020	206M^a^
YouthBio Therapeutics	2020	<10M
Retro Biosciences	2021	180M^a^
Altos Labs	2021	3B
New Limit	2021	105M

^a^ Subsidiary/working on other programs besides reprogramming.

## 10 LIMITATIONS AND FUTURE PERSPECTIVES FOR IVPR

Multiple publications on partial reprogramming have confirmed a wide variety of restorative effects on lifespan, epigenetic age, aging hallmarks, and tissue regeneration, yet hurdles to successful clinical translation remain (Goya et al., 2018; Singh & Zhakupova, 2022). In order to specifically address these hurdles, such as teratoma formation or context dependent effects, a better understanding of the mechanisms and control over the reprogramming process combined with technical advances in delivery and targeted expression are required.

Developmental models both highlight and provide some insights to address these limitations. For example, during development, cellular reprogramming in mice is a direct and efficient process with a well‐orchestrated gain of pluripotency after only 3.5 days (Paranjpe & Veenstra, 2015). Similarly, SCNT also follows a relatively direct reprogramming process driven by maternal factors (Han et al., 2015; Jullien et al., 2011). Alternately, current transcription factor based reprogramming rejuvenation methods follows a stochastic process that suffer from the inability to universally reprogram target cells along the same trajectory at the same rate and to the same degree in a timely manner. Further delineation of the barriers specific to subpopulations within a tissue that result in this asynchronous process with altered reprogramming potential are needed. One way to address this problem was demonstrated by the Brunet laboratory in 2019. Using a single‐cell RNA‐seq and multi‐omic profiling approach, they identified key contributors to reprogramming variability in aged fibroblasts such as inflammatory cytokines (Mahmoudi et al., 2019). At the same time, groups have used a bioinformatic approach to mine reprogramming data sets in order to enable identification of shared gene targets and enriched cellular pathways that could be utilized to develop enhanced reprogramming (Kareta, 2016; Knyazer et al., 2021). In this line, Calico recently performed a screening of reprogramming factors to better characterize reprogramming induced rejuvenation while simultaneously identifying a novel multipotent strategy derived from amphibian regeneration and the MSX1 transcription factor (Roux et al., 2022). Performing screenings via RNA interference, CRISPR knockdowns, or reprogramming compound libraries also present a viable way forward to better understand and optimize cellular reprogramming (Lee et al., 2019). Together, these examples demonstrate the innovative routes and improved methods of reprogramming needed in order to allow the development of future clinical translation.

An optimized and synchronous process of cellular reprogramming will only exacerbate the need for improved tunability and delivery to avoid deleterious effects. Consequently, cell‐specific reprogramming via targetable delivery vectors might be the next step for a safe and effective clinical therapy. Currently, several groups have demonstrated translational methods of ectopic factor expression utilizing either naked plasmid DNA in situ, localized AAV injection, or ex vivo modified mRNA transfection to induce rejuvenation (de Lázaro et al., 2019; Lu et al., 2020; Sarkar et al., 2020). Although promising, in certain cases these methods can suffer from poor delivery efficiency, immunogenicity, and poor specificity. Clinical use of gene therapy has surged over the last couple decades^100^. Interesting improvements in next generation gene insertion strategies and RNA gated payload expression could be potential game changers for the field, although they also require improvements to delivery (Burgess, 2022; Jiang et al., 2022; Srivastava & DeWitt, 2016; Yarnall et al., 2022). In several cases, groups have observed effective delivery using a variety of different nanoparticles for in vivo reprogramming (Ofenbauer & Tursun, 2019; Romanazzo et al., 2020). Preliminary work from Huang et al., Tang et al., and our laboratory (under review) have demonstrated treatment with reprogramming small molecules can enhance regeneration in vivo, induce multiparameter rejuvenation in vitro, and extend lifespan in *C. elegans* (Huang et al., 2018; Schoenfeldt et al., 2022; Tang & Cheng, 2017). For these reasons, chemical induced partial reprogramming (ciPR) could be a particularly intriguing method of IVPR, especially if combined with prodrug targeting strategies such as antibody directed enzyme prodrug therapy (ADEPT; Sharma & Bagshawe, 2017; Tang & Cheng, 2017).

In summary, the partial reprogramming data reviewed here present a currently limited rejuvenation strategy capable of ameliorating aging phenotypes, increasing healthspan or lifespan, restoring epigenetic age, and improving tissue regeneration and tissue function (Tables 1, 2, 3 and 4). As detailed above, specific cases only show limited or no improvements across similar aging readouts and models, potentially due to the stochastic nature of reprogramming along with cell intrinsic and microenvironment dependent effects. To address these lingering questions, improvements in mechanistic understanding, delivery, safety, tunability, and efficiency will be required. Though the challenges for this endeavor are considerable, collective improvements could pave the way to a future where effective treatment of AADs is attainable and hopefully no longer a serious burden to society.

## AUTHOR CONTRIBUTIONS

P.T.P., A.N., and A.O. conceived the idea for the review. P.T.P. and A.N. wrote the manuscript and created the figures and tables with contributions and editing from A.O.

## ACKNOWLEDGEMENTS

We would like to acknowledge all members of the Ocampo laboratory for support during writing.

## FUNDING INFORMATION

No funding information provided.

## CONFLICT OF INTEREST STATEMENT

A.O. is co‐founder and shareholder of EPITERNA SA (non‐financial interests) and co‐founder of Longevity Consultancy Group (non‐financial interests). The rest of the authors declare no competing interests.
